# The Relationship between Football Participation and Sport Trait Confidence for Adolescents: A Chain-Mediated Effect of Collective Self-Esteem and Self-Esteem

**DOI:** 10.3390/bs14080656

**Published:** 2024-07-31

**Authors:** Deyang Yu, Xiang Che, Haopeng Wang, Ning Ma, Zicheng Wan, Bingjun Wan, Xuqun You, Kun Zhang, Nian Li, Yizhou Shui

**Affiliations:** 1 School of Physical Education, Shaanxi Normal University, Xi’an 710119, China; yudeyang@snnu.edu.cn (D.Y.);; 2 School of Psychology, Shaanxi Normal University, Xi’an 710061, China

**Keywords:** sports participation, collective self-esteem, self-esteem, sport trait confidence, adolescents, football

## Abstract

To investigate the relationship between sports participation and sport trait confidence, 1659 teenagers in primary and secondary schools who regularly play football were asked to complete the Sports Participation Scale, Collective Self-Esteem Scale, Self-Esteem Scale, and Sport Trait Confidence Scale. The results show that (1) the positive prediction of football participation on sport trait confidence is not significant; (2) collective self-esteem and self-esteem play a mediating role between football participation and sport trait confidence; (3) the mediating effect occurs through three pathways. The study provides theoretical guidance and empirical evidence for the lead and intervention of adolescent football participation on sport trait confidence. This study created a chain-mediated model to examine the mediating role of collective self-esteem and self-esteem in their relationship, as well as the impact of the two as chain mediators on football participation and sport trait confidence.

## 1. Introduction

Research has pointed out that sports participation refers to physical and mental engagement in physical education classrooms or extracurricular sports activities, usually divided into three dimensions: cognitive participation, emotional participation, and behavioral participation [1].

Collective self-esteem refers to the positive self-concept derived from one or more social groups, which is an evaluation and perception of the social group to which one belongs. It mainly includes three parts: the value of the collective itself, the individual’s perception that the group to which they belong is respected, and the value that the collective bestows upon the individual [2].

The concept of self-esteem was first introduced into psychology by experts focusing on functionalist psychology [3]. This study defines self-esteem as the evaluation of an individual’s own value and abilities during the process of socialization [4].

Researchers generally believe that during exercise, self-confidence can be divided into two types: sport trait confidence and sport state confidence. Sport trait confidence refers to the belief or degree of certainty that one has the ability to succeed in sports at a specific moment. Sport state confidence is the belief or degree of certainty that one has the ability to succeed in a sport at a given moment in time [5].

During participation in football, self-esteem, as an internal psychological state, is an important factor in promoting sports participation among teenagers [6]. Some scholars believe that self-esteem has a promoting effect on sports participation behaviour, and self-esteem is also one of the motivations for promoting sports participation. To improve the participation of college students in sports, we should pay attention to the cultivation of college students’ self-esteem [7]. Research has shown that the more time children spend on team sports, the more confidence they will have in their athletic abilities, which is positively correlated with self-esteem [8]. Some findings indicated that children who spent more time in team sports, but not time in individual sports, reported higher self-esteem [9]. Therefore, football as a standard team sport may have a more significant impact.

Football training has different effects on different subjects. With age growth and years of systematic training, athletes not only improve their athletic level, but also enhance their psychological adaptability and corresponding psychological self-control. They can better grasp the psychology of participating in various competitions, and their understanding of basic skills and tactics, as well as their familiarity with major competitions, make them more confident and tend to be stable. According to a questionnaire survey, Peng Maofa [10] found that children’s ability to analyze and solve problems is greatly improved after participating in football training. When facing difficulties, they can think more independently and no longer rely on their parents. From these findings, it can be seen that the confidence brought about by football training and games not only affects the performance and coping ability of athletes on the field, but also helps teenagers to deal with problems in daily life.

As a team sport, football plays an important role in collective self-esteem. By participating in football, teenagers can establish connections with others and enjoy the fun of sports together. This mutual assistance helps to enhance a sense of belonging, thereby improving an individual’s collective self-esteem [11]. Through research on extracurricular collective activities in universities, it was found that those who have participated in collective activities have better collective self-esteem, a higher sense of belonging to the campus, and a higher average score [12]. Based on this, collective self-esteem may be enhanced when participating in collective sports and gaining recognition within the team, and it can affect the overall performance of the team.

Collective self-esteem and individual self-esteem are two concepts that differ in their sources, focus, and psychological impact. When an individual is able to hold a positive evaluation of the social group to which they belong, their acceptance and sense of self-worth will increase [13]. Some researchers suggested collective self-esteem can have a positive effect on individual self-esteem. This is because an improvement in collective self-esteem can enable individuals to have a correct understanding and reasonable evaluation of themselves in the group, thereby improving individual self-esteem [2]. As team sport players, football players ‘collective self-esteem will be enhanced with their participation in this sport, external recognition, fan promotion, and public recognition. It will further enhance individual self-esteem [14]. Ultimately, individual sport trait confidence may be fed back to team and game performance, but the specific path and mechanism need to be studied.

The main objective of this study was to explore whether collective self-esteem and self-esteem play a chain-mediating role between sports participation and sports trait confidence. The specific research hypotheses are as follows:

**H1.**  *Collective self-esteem and self-esteem can act as chain mediators to influence football participation and sport trait confidence.*

**H2.**  *There is a positive predictive relationship between football participation and sport trait confidence.*

**H3.**  *Self-esteem can act as a mediator to influence football participation and sport trait confidence.*

**H4.**  *Collective self-esteem can act as a mediator to influence football participation and sport trait confidence.*

### The Present Study

Some studies found that players with similar technical levels exhibit vastly different levels of competitiveness in matches, and even the same player’s level of competitiveness varies greatly in different matches [15]. In previous studies, external factors, such as the levels of opponents and teammates, as well as the tactical arrangements of coaches, have been proven to affect the performance of players [5]. Some studies have also explored the impact of self-esteem and sport trait confidence on athletic performance from the perspective of players. With the deepening of research, the impact of sports participation on players themselves has received widespread attention. Therefore, it is necessary to explore the impact and mechanism of sports participation on sport trait confidence, as well as the mediating role of collective self-esteem and self-esteem in it.

## 2. Methods

### 2.1. Design

We have selected authoritative scales in the field of psychology based on our needs and have made adjustments for specific populations and situations on this basis. For example, in the Sport Participation Scale, we changed participation in physical education classes to participation in football training. The specific selection of scales is shown in the following text.

#### 2.1.1. Collective Self-Esteem Scale

The Collective Self-Esteem Scale developed by Luhtanen [2] was adopted, which was locally revised and translated into a Chinese version by Zhang [16]. This scale includes four dimensions: member self-esteem, internal collective self-esteem, external collective self-esteem, and identification influence. There are 16 items, and a 7-point scoring system is used, ranging from 1 (completely disagree) to 7 (completely agree). Among them, 1, 3, 6, 8, 9, 11, 14, and 16 are positive scores, and the other 8 items are negative scores. Cronbach’s α coefficient during this survey was 0.808 [17].

#### 2.1.2. Sport Trait Confidence Scale

The Sport Trait Confidence Scale 1 compiled by Vealey [5] was adopted. The scale was locally revised and translated into a Chinese version by Zhang [18]. A total of 13 entries were included, using a 9-point scoring system, ranging from 1 (low) to 9 (high). All entries are scored in positive terms. Cronbach’s α coefficient during this survey was 0.953.

#### 2.1.3. Self-Esteem Scale

Rosenberg’s Self-Esteem Scale [19] was adopted, which was locally revised and translated into a Chinese version by Wang [20]. There are 10 items, and a 4-point scoring system is used, ranging from 1 (completely inconsistent) to 4 (completely consistent). Among them, 3, 5, 8, 9, 10 are positive scores, and the other 5 entries are reverse scores. Cronbach’s α coefficient at the time of this survey was 0.823.

#### 2.1.4. Sports Participation Scale

The Sports Participation scale was developed by Agbuga [21], and it was localized and revised and translated into a Chinese version by Zhang [22]. The version includes three dimensions: behavioral participation, emotional participation, and cognitive participation. There are a total of 13 items, with a 5-point scoring system ranging from 1 (fully compliant) to 5 (completely non-compliant). Cronbach’s α coefficient during this survey was 0.971.

### 2.2. Setting

The recruitment phase and data collection were completed from the 10th to 15th of December 2023, and, ultimately, 64 schools registered to participate in this study. We distributed and filled out scales in pre-prepared classrooms and collected data on site.

### 2.3. Participants

The participants we selected were teenagers aged 8–16 who regularly participated in football training and love football. Other students who do not play football were not included in the scope. They all participated in after-school training every day with the school football team.

A total of 1692 participants from 64 schools across 5 provinces in China were selected. We collected data from 30 primary schools and 18 secondary schools. After screening, 1659 valid questionnaires were obtained (Table 1), resulting in a 97% validity rate. The participating schools provided formal consent approved by the parents prior to filling out the questionnaires. The methods for gathering data respected participants’ rights and followed ethical guidelines. This study was approved by the Ethics Committee of Shaanxi Normal University (NO. 202416013)

### 2.4. Procedures

After being informed of the purpose of the questionnaire and the security of the data information, the participants filled out 4 forms, which were the Collective Self-Esteem Scale, Sports Trait Confidence Scale, Self-Esteem Scale, and Sports Participation Scale in a random order. Finally, students reported demographic information, such as gender, age, and education level. Explanation and anonymity, no right or wrong answers, and so on were included.

### 2.5. Outcome Measures

All data were statistically analyzed using SPSS 27.0. Firstly, the original data was preliminarily input and analyzed. The missing values in the data were replaced using the sequence mean interpolation method, and extreme data were removed. The reliability and validity of the questionnaire were tested using SPSS 27.0. Then, all data were standardized and correlated. The chain-mediated model was tested using the PROCESS program Model 7 [23]. The significance of the mediation effect was tested using the bias corrected percentile bootstrap method.

### 2.6. Bias

Our sample selection is comprehensive, including cities and towns with different economic development situations, as well as five cities in different areas.

We also conducted on-site supervision to minimize data bias. Furthermore, in the subsequent data processing, questionnaires with overly consistent answers were excluded.

### 2.7. Analysis

Due to the use of questionnaires for data collection in this study, exploratory factor analysis was used to test for possible common method biases. Using Harman’s single-factor test method [24], the results showed that 7 factors had eigenvalues greater than 1, with a total of 52 factors inputted. The first factor extracted explained a variation of 26.697%, which was less than the critical value of 40%. Therefore, there are no significant common method biases in the data of this study.

The descriptive statistical analysis of each variable is shown in Table 2, and there is a significant positive correlation between sports participation as an independent variable and collective self-esteem (r = 0.207, *p* < 0.001), self-esteem (r = 0.223, *p* < 0.001), and sport trait confidence (r = 0.146, *p* < 0.001). Collective self-esteem is significantly positively correlated with self-esteem (r = 0.591, *p* < 0.001) and sport trait confidence (r = 0.527, *p* < 0.001), and self-esteem is also significantly correlated with sport trait confidence (r = 0.518, *p* < 0.001).

The results of the mediation effect analysis (Table 3) indicate that sports participation has a positive predictive effect on self-esteem (β = 0.106, *p* < 0.001) and collective self-esteem (β = 0.207, *p* < 0.001). However, the predictive effect of sports participation on sport trait confidence (β = 0.006, *p* = 0.785) is not significant. Collective self-esteem can positively predict self-esteem (β = 0.569, *p* < 0.001) and sport trait confidence (β = 0.339, *p* < 0.001). Self-esteem can positively predict sport trait confidence (β = 0.316, *p* < 0.001). Therefore, the mediating variables acted as full mediators, i.e., collective self-esteem and self-esteem played a full mediating role in the effect of football participation on sport trait confidence.

**Figure 1:**
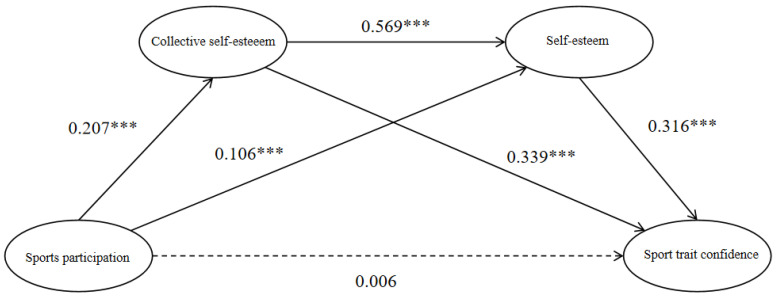
The chain-mediated model, *** *p* < 0.001.

The mediation effect analysis results are shown in Table 4 and Figure 1. The mediation effect of collective self-esteem and self-esteem is significant, and the mediation effect is generated through three mediation chains: first, sports participation → collective self-esteem → sport trait confidence (Ind1), with a 95% confidence interval of (0.070, 0.146), indicating that collective self-esteem mediates sports participation and sport trait confidence; second, sports participation → self-esteem → sport trait confidence (Ind2), with a 95% confidence interval of (0.028, 0.076), indicating that self-esteem mediates sports participation and sport trait confidence; third, sports participation → collective self-esteem → self-esteem → sport trait confidence (Ind3), with a 95% confidence interval of (0.037, 0.078), indicating that collective self-esteem and self-esteem chain-mediate sports participation and sport trait confidence.

## 3. Discussion

The research results indicate that participation in football has an association on sport trait confidence through the mediating effect of collective self-esteem and self-esteem. The mediating effect occurs through three pathways, including the independent mediating effect of self-esteem and collective self-esteem and the chain-mediated effect of collective self-esteem and self-esteem. Although there is indeed a positive relationship between sports participation and sport trait confidence, the predictive effect is not significant.

This study found that both collective self-esteem and self-esteem can act as mediators to influence sport participation and sport trait confidence; as such, H1 is supported. Collective self-esteem and self-esteem have a certain impact on the motivation of adolescents to exercise. Over time, higher self-esteem among adolescents predicted higher levels of sports participation [18]. Collective self-esteem affects an individual’s psychological state and self-esteem and ultimately feeds back to sport trait confidence and performance on the field. It forms a virtuous cycle. The chain-mediated effect of collective self-esteem and individual self-esteem on sports participation and sport trait confidence is manifested at multiple levels. Firstly, they can enhance the intrinsic motivation of athletes, making them more passionate about sports and willing to invest more time and energy [25]. Secondly, they can strengthen athletes’ ability to set goals and achieve them, thereby enhancing their performance and satisfaction [26]. Finally, collective and individual self-esteem can also help athletes better cope with stress and setbacks, maintain a positive attitude, and perform at a high level [27].

This study found that there is no significant positive predictive effect between football participation and sport trait confidence. Therefore, H2 is not supported. Some scholars believe that sports can cultivate self-confidence in nine ways, including the sense of achievement gained in team sports and sufficient personal self-esteem [28]. By playing football, teenagers can have a better understanding of themselves and enhanced self-confidence with a positive self-image [29].

As such, based on existing studies, we speculate that football participation is likely to have an impact on sport trait confidence through the mediating effect of collective self-esteem and self-esteem. It is not just that participating in football can affect the sport trait of confidence; it is also necessary to positively cultivate the sense of gain in the team and the personal self-esteem of teenagers. This further illustrates the role of this study in cultivating youth football and confidence, providing direction and guidance for the teaching of physical education teachers, and the development of young people themselves.

This study found that collective self-esteem can act as a mediator to influence sport participation and sport trait confidence, meaning that H3 is supported. As a team sport, football collective self-esteem can strengthen cohesion among team members [30]. When team members have a high sense of self-esteem towards the team, they are more likely to actively participate in training and competitions because they feel that they are an important part of the team [31]. Confident teenagers often perform better in competitions, and this positive psychological state can help them stay calm at critical moments, thus achieving better results.

Football has unique advantages in promoting the physical, psychological, and social abilities of adolescents [32]. Understanding the impact of collective self-esteem on sports participation and sport confidence can help coaches and educators to design more effective psychological interventions to promote the overall development of adolescents. Campus football culture and team culture have a significant impact on collective self-esteem. A positive, encouraging, and supportive campus culture can promote the collective self-esteem of adolescents, thereby enhancing their sport trait confidence [33]. Therefore, focusing on the improvement of collective self-esteem is beneficial for enhancing the sport trait confidence of adolescents. Individual self-esteem has been shown to affect team performance in sports, and the level of self-esteem in sports teams is influenced by the type of sports they engage in. Compared to participating in individual sports, adolescents who participate in collective sports exhibit higher collective self-esteem [13].

This study found that self-esteem can act as a mediator to influence sport participation and sport trait confidence, meaning that H4 is supported. Sport participation has a significant impact on self-esteem and collective self-esteem, which is consistent with previous research. Fox [34] analyzed 80 studies and found that sport participation can explain approximately 50% of the variance in collective self-esteem, indicating that sport participation is an effective medium for promoting collective self-esteem.

Sonstroem [35] proposed a model based on the multidimensional hierarchical self-esteem theory, which explores the effects of sport participation on individual and collective self-esteem and further reveals the mechanisms by which exercise participation affects individual and collective self-esteem. The model suggests that self-concept in some subdomains (such as physical, cognitive, and social domains) constitutes the collective self-esteem of a team, while specific domains at the lowest level of the model constitute each sub-domain. High levels of collective self-esteem increase with the level of self-esteem in specific areas at lower levels and vice versa. Sports participation can promote the self-esteem of adolescents, and the improvement of self-esteem levels helps to enhance the collective self-esteem levels of adolescents.

Self-esteem is a person’s evaluation of their own value and abilities, and it plays an important role in an individual’s mental health and social adaptation. In the field of sports, self-esteem is also considered to be an important factor affecting athletes’ participation in sports and improving their sport trait confidence [36].

Self-esteem can affect an individual’s participation in sports, which is consistent with previous research findings. Individuals with high self-esteem are usually more confident in facing challenges and willing to try new sports, while individuals with low self-esteem may avoid participating in sports due to a fear of failure or being evaluated by others. In addition, individuals with high self-esteem are more likely to persist when facing difficulties in doing sports, while individuals with low self-esteem may easily give up [37].

Secondly, self-esteem can also affect an individual’s sport trait confidence. Individuals with high self-esteem usually have a higher evaluation of their abilities, so their performance in sports is more likely to receive recognition from others, therefore improving their sport trait confidence. On the opposite, individuals with low self-esteem may have doubts about their abilities, which can affect their performance in sports and lower their sport trait confidence [27].

As a consequence, self-esteem, as a mediator, can influence sports participation and sport trait confidence. In order to enhance individual sport participation and sport trait confidence, attention needs to be paid to the self-esteem of adolescents [38].

The aim of this study is to explore the impact of adolescent participation in football on their sport trait confidence, as well as the roles and mechanisms played by self-esteem and collective self-esteem. By playing football, teenagers can better understand themselves, establish a positive self-image, and, therefore, enhance their self-confidence [39]. Meanwhile, participating in football often requires teamwork, which helps teenagers learn how to communicate and collaborate with others, and to improve their interpersonal skills. High levels of sport trait confidence have a significant impact on football players’ competitive performance [38]. Related studies have found that higher sport trait confidence can improve adolescent sports performance, and the improvements in self-esteem brought about by sports performance can have an impact on sport trait confidence [40].

The goal of playing football is not only physical exercise, but also comprehensive attention to the physical and mental health of students, which can help teenagers to achieve balanced development in multiple aspects, such as physical and psychological well-being [41]. This theory can also directly guide football educators to pay more attention to student participation in sports rather than simply teaching daily physical practice. It also promotes the requirements of the current role transformation of football educators, providing theoretical support for future football teaching activities.

## 4. Limitations and Strengths

Firstly, this study is a cross-sectional study, lacking longitudinal examination and monitoring, which to some extent limits the causal inference and application promotion of the research results. In order to further reveal the hierarchical relationship and degree of correlation among sports participation, collective self-esteem, self-esteem, and sport trait confidence, future research can consider using the longitudinal multi-period, multi-region, and multi-population data acquisition methods. By conducting cross-lagged tests, exploring multi-level models, and designing variables, such as mediators and moderators, we can gradually examine the complex relationships among these variables, providing a scientific basis for formulating more effective prevention and intervention measures. Follow-up studies can include a control group to compare with other sports. This study explores the mechanism of collective self-esteem and self-esteem as a mediator on sports participation and sports trait confidence and provides theoretical guidance for teachers and coaches. While encouraging young people to participate in football, we should pay attention to the cultivation of collective self-esteem and self-respect.

## 5. Conclusions

This study created a chain-mediated model to examine the mediating role of collective self-esteem and self-esteem in their relationship, as well as the impact of the two as chain mediators on football participation and sport trait confidence. This study suggested that football participation can promote higher sport trait confidence through higher collective self-esteem or self-esteem. High levels of collective self-esteem and self-esteem in football participation can work together to create high levels of sport trait confidence. Adolescents should pay more attention to the development of collective self-esteem and self-esteem when playing football with their friends.

## Figures and Tables

**Table 1:** Demographic characteristics of participants.

Variables	Numbers	Percentages	Variables	M ± SD
Gender				
**Male**	1135	68.415%	Weekly training hours (by hours)	
**Female**	524	31.585%	Primary	6.986 ± 4.851
Phase of study			Junior high	7.007 ± 4.846
**Primary**	885	53.345%	Senior high	7.467 ± 4.949
**Junior high**	543	32.731%	Training years (by years)	
**Senior high**	231	13.924%	Primary	3.853 ± 2.387
Place of abode			Junior high	3.870 ± 2.391
**City**	1398	84.268%	Senior high	4.158 ± 2.513
**Village**	261	15.732%		

**Table 2:** Correlation analysis.

Variables	M	SD	1	2	3	4
1. Sports Participation	4.089	0.781	1			
2. Collective Self-Esteem	5.252	0.740	0.207 ***	1		
3. Self-Esteem	3.158	0.506	0.223 ***	0.591 ***	1	
4. Sport Trait Confidence	7.016	1.437	0.146 ***	0.527 ***	0.518 ***	1

Note: The mean (M) and standard deviation (SD) are calculated by dividing the total score by the individual item of the scale, *** *p* < 0.001.

**Table 3:** Regression analysis between variables.

Outcome	Predictive Variables	R^2^	F	β	t
Collective Self-Esteem	Sports Participation	0.042	73.992	0.207	8.602 ***
Self-Esteem	Sports Participation	0.359	465.005	0.106	5.264 ***
	Collective Self-Esteem			0.569	28.301 ***
Sport Trait Confidence	Sports Participation	0.342	287.985	0.006	0.273
	Collective Self-Esteem			0.339	13.664 ***
	Self-Esteem			0.316	12.695 ***

Note: *** *p* < 0.001.

**Table 4:** Test of the mediating effect.

Path	Effect Value	SE	Bootstrap 95% CI		Relative Mediating Effect
Ind1	0.105	0.020	0.146	0.070	49.905%
Ind2	0.051	0.012	0.076	0.028	24.240%
Ind3	0.056	0.011	0.078	0.037	26.616%

Note: The boot standard error, boot CI lower limit, and boot CI upper limit refer to the standard error, 95% confidence interval lower limit, and upper limit of the indirect effects estimated using the bias corrected percentile bootstrap method, respectively. All numerical values are rounded to two decimal places.

## Data Availability

The data that support the findings of this study are available from the corresponding author upon reasonable request.
